# The phylogeographic journey of a plant species from lowland to highlands during the Pleistocene

**DOI:** 10.1038/s41598-024-53414-4

**Published:** 2024-02-15

**Authors:** Luana Sousa Soares, Loreta B. Freitas

**Affiliations:** Department of Genetics, Universidade Federal do Rio Grande do Sul, PoBox 15053, Porto Alegre, 91501-970 Brazil

**Keywords:** Population genetics, Evolutionary genetics, Adaptive radiation

## Abstract

Phylogeographic history refers to how species evolve and diversify in response to historical, ecological, and demographic factors. The climate fluctuation during the Pleistocene period marked a crucial time in shaping many species’ distribution and genetic structure, particularly those from southern South American grasslands. This work investigated the phylogeographic history of a highland grassland, *Petunia altiplana* T. Ando & Hashim. (Solanaceae), its diversity, and geographic distribution using a population genomic approach based on RAD-seq data. Our results indicated that, during the Pleistocene, when the grasslands expanded to highlands, the lowland populations of *P. altiplana* reached the higher open fields, enlarging their geographic distribution. We found that the *P. altiplana* genetic diversity followed the geographic division into eastern (E) and western (WE) population groups, with a subtle division in the E group regarding the Pelotas River headwater. The results also showed that isolation by distance was the main divergence pattern, with elevation playing a pivotal role in shaping WE and E groups. Our findings indicated that lowland-adapted populations quickly colonized highlands during the late Pleistocene*.*

## Introduction

The reduction in gene flow between populations and lineages due to multiple processes can lead to genetic differentiation and impact the demographic history of a species^1^, with isolation by distance (IBD)^2^ isolation by altitude (IBA)^3^, and isolation by environment (IBE)^4^ playing significant roles.

The Pleistocene period, with its climate changes alternating warm and wet and cold and dry cycles between ~ 2.5 million years ago (Mya) and ~ 11 thousand years ago (Kya), was one of the most significant historical events that influenced species’ distribution and structure around the World^5^. In South America, climatic shifts profoundly impacted many species, forming refugia in tropical forests^6^ and grasslands^7^. Quaternary climate cycles affected narrowly and widely distributed species, with grassland-adapted species expanding while forests contracted^8^. The grassland expansion in the highlands in southern South America persisted for a long time. In contrast, their contraction only occurred during the Holocene (~ 4 Kya)^9^, concomitantly with the *Araucaria* Forest expansion. Such oscillations have drastically transformed the landscape and species distribution. During forest expansion, grasslands were fragmented and confined to higher elevations, isolating populations in sky-island patches surrounded by forest^10^. Such isolation allowed allopatric speciation in many plant genera^11,12^, with multiple examples of similar diversification and distribution patterns for different organism groups inhabiting the highland grasslands^13,14^.

The genus *Petunia* (Solanaceae) has been included in genetics and molecular biology studies and is a valuable model for eudicots^15^. Similarly, these South American native herbs may help understand the evolutionary aspects of plant speciation under a phylogeographical approach. The most inclusive phylogenetic analysis^16^ indicated that the genus originated in lowland grasslands in southern South America, from where it dispersed to highland fields during the Pleistocene. The genus diversified between 1.3 Mya^17^ and 2.8 Mya^18^, and ecological interactions such as climate, soil, and pollinators have strongly influenced speciation^19^. The Pleistocene climate changes have also played a role in the differentiation of intraspecific lineages, favoring the expansion and colonization of new environments^20^. Studies on population structure and diversity of lowland and coastal *Petunia* species are predominant, whereas just a few focus on the highland lineages^11,17^. Such highland species are endemic, and each occupies a narrow range in elevation.

**Figure 1:**
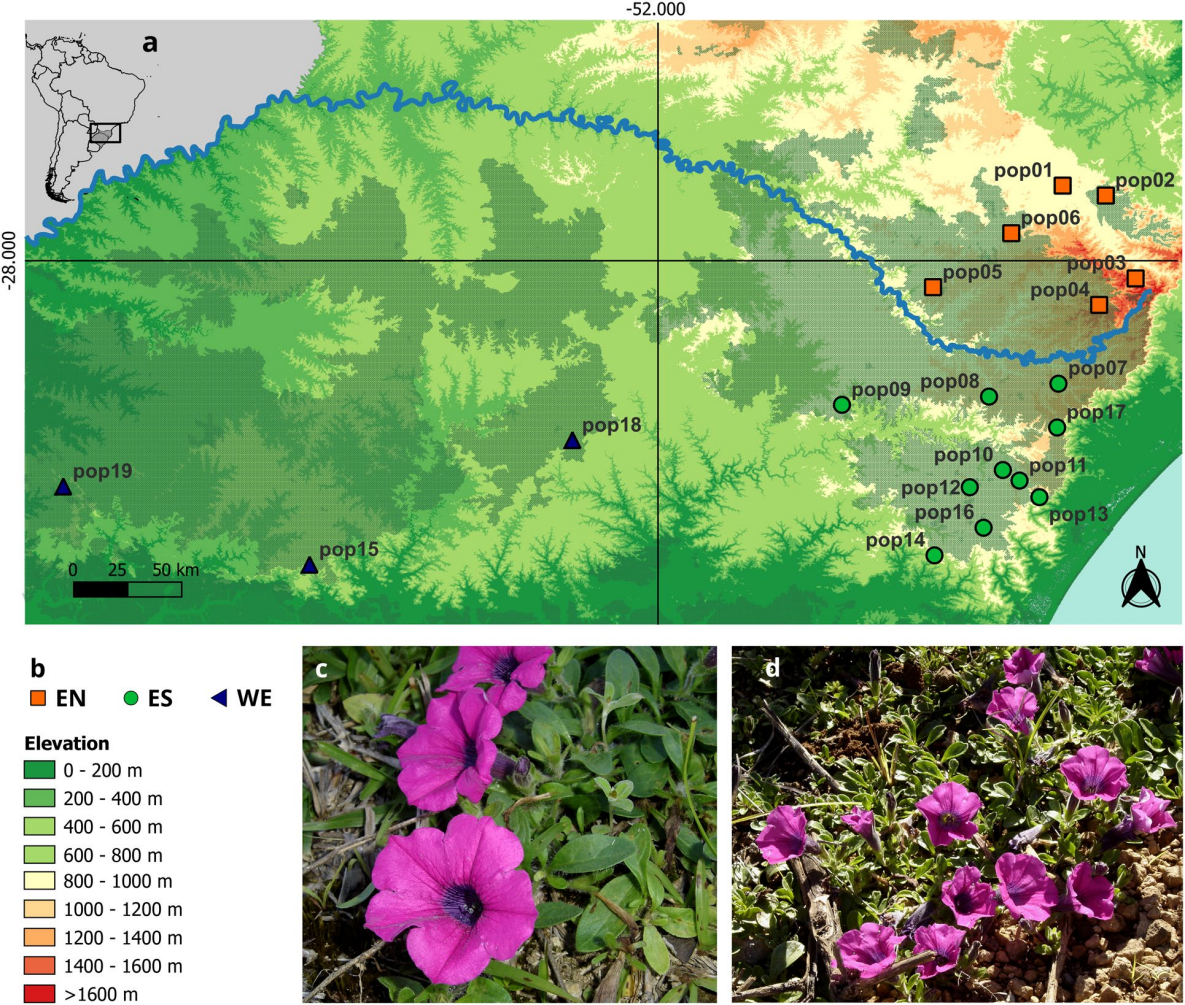
Geographical distribution of *P. altiplana*. (A) Location of collection sites (populations). Colors indicate the population groups: orange squares, EN; green circles, ES; and blue triangles, WE. (B) Elevation indicators. (C) Floral morphology of *P. altiplana* (D) and general view of the flowers (photos J.R. Stehmann, Universidade Federal de Minas Gerais, Brazil). The checkered green pattern indicates the distribution of the southern Brazilian grassland^24^—a mosaic landscape between the Atlantic Forest and Pampa domains.

Here, we studied the widely distributed *Petunia altiplana* T. Ando & Hashim. species, which occupies the southern Brazilian subtropical highland grasslands (SHG). SHG is the border between two distinct domains, the Atlantic Forest and Pampas^21^ (Fig. 1). Despite the large area and elevation range [~ 400 to 1400 m above sea level (m a.s.l.)], *P. altiplana* individuals are found in sparse patches throughout the species’ distribution, growing on rocky outcrops and roadside slopes^22^. The *P. altiplana* geographical range is naturally fragmented due to the region’s phytophysiognomy, which consists of a mosaic between grasslands and *Araucaria* Forest^23^ and the Pelotas River course. Moreover, anthropic activities have severely impacted the highland grasslands, which affected the native species’ distribution^24^. Estimates on intraspecific diversity based on plastid sequences revealed two main population groups in *P. altiplana*, with the Pelotas River separating them^17^. The haplogroups were mutually exclusive regarding each riverbank, and the authors considered the Pelotas River a phylogeographic barrier. In turn, analyses based on nuclear microsatellites^24^ indicated moderate bidirectional gene flow between the riverbanks, smaller than observed between populations from the same side of the Pelotas River. This last work^24^ also included plastid haplotypes for more populations and individuals than the previous work^17^, recovering the two haplogroups regarding riverbanks, with three low-frequent new haplotypes shared between populations from the two river’s margins.

Plastid markers and microsatellites are known to be less informative in understanding population genetics^25^ compared with RAD-seq-derived data. Moreover, plastid sequences and nuclear microsatellites often result in different evolutionary patterns due to discrepancies in coalescence time^26^. So, to estimate the *P. altiplana* genetic diversity and disentangle its population structure and demography, we used a next-generation sequencing method to investigate the factors that have influenced the species’ phylogeographic history. With this analysis, we aimed to answer the questions: (1) What is the demographic history of the species? (2) What are the primary factors that influenced the species structuration? (3) Are the populations structured due to the Pelotas River course or other physical or ecological barriers?

## Results

### Quality of genomic data and SNP calling

We obtained 186,032,269 raw reads considering all individuals. Reads ranged between 766,718 to 3,342,929 per individual, averaging 1,788,711.82. We eliminated 771,892 loci that did not meet population constraints and retained 18,612 loci with 1,288,087 sites, which produced a matrix with 11,231 variants (Supplementary Information 1 and 2: Tables S1 and S2). The average number of genotyped sites per locus in STACK after the filtering was 69.21 base pairs (bp) [standard deviation (SD) 0.01]. We removed 463 outlier loci, resulting in a final dataset containing 10,768 potentially neutral SNP used in the subsequent analyses.

### Genetic diversity and population structure

Nucleotide diversity (π) varied between 0.15 and 0.20 across the collection sites. In all localities, the inbreeding coefficients were low (*F*_IS_ < 0.1), with pop01 and pop19 having the lowest values (Table 1). Pop19 had the higher number of private alleles (PA) among populations, ca. four times higher than the second population (pop6). Pop19 is the most distantly distributed population regarding remains.

**Table 1:** Sampling information and diversity indices of *Petunia altiplana*. Diversity indices were estimated per population and population groups.

Sampling information							Diversity indices							
Group	Pop ID	Long	Lat	Elevation	Voucher	N	Private	SNPs	P	%P	H_E_	H_O_	π	*F*_IS_
EN	pop01	− 49.9667	− 27.6000	839	BHCB 80,059	4	11	10,680	4852	38%	0.16	0.18	0.17	− 0.02
	pop02	− 49.7500	− 27.6500	893	BHCB 99,674	5	4	10,679	5413	43%	0.17	0.16	0.20	0.08
	pop03	− 49.6000	− 28.0667	1404	BHCB 96,683	5	60	10,674	4307	34%	0.14	0.16	0.16	0.01
	pop04	− 49.7833	− 28.2000	1424	BHCB 99,752	5	4	10,683	4772	37%	0.15	0.15	0.17	0.04
	pop05	− 50.6178	− 28.1107	976	NA	4	1	10,669	4524	36%	0.15	0.15	0.18	0.06
	pop06	− 50.2249	− 27.8393	929	BHCB195626	5	70	10,683	5604	44%	0.16	0.16	0.17	0.02
	**EN**					28	797	10,683	8937	86%	0.17	0.17	0.20	0.15
ES	pop07	− 49.988	− 28.596	1130	BHCB104859	5	1	10,677	4600	36%	0.14	0.13	0.17	0.08
	pop08	− 50.336	− 28.661	1064	BHCB195625	1	0	9193	1246	11%	0.07	0.13	0.13	0.00
	pop09	− 51.076	− 28.702	844	NA	4	10	10,671	4278	34%	0.14	0.15	0.17	0.04
	pop10	− 50.267	− 29.030	955	BHCB116998	3	0	10,671	3908	31%	0.13	0.15	0.17	0.03
	pop11	− 50.183	− 29.083	930	BHCB 79,907	6	3	10,683	5158	41%	0.15	0.15	0.17	0.04
	pop13	− 50.083	− 29.167	946	BHBC 87,269	5	0	10,683	4820	38%	0.15	0.15	0.17	0.04
	pop14	− 50.611	− 29.458	920	BHCB 79,906	5	5	10,680	4649	36%	0.14	0.15	0.16	0.02
	pop16	− 50.364	− 29.319	969	BHCB195621	4	1	10,678	4116	32%	0.14	0.16	0.16	0.08
	pop17	− 49.994	− 28.816	1113	BHCB195623	6	2	10,682	5436	43%	0.16	0.12	0.17	0.05
	**ES**					39	547	10,683	8753	87%	0.15	0.15	0.19	0.17
WE	pop15	− 53.750	− 29.505	443	BHCB195622	3	7	10,613	3409	29%	0.13	0.15	0.16	0.00
	pop18	− 52.430	− 28.880	647	BHCB114597	3	4	10,562	4116	26%	0.12	0.11	0.15	0.06
	pop19	− 54.988	− 29.113	371	BHCB201021	4	259	10,644	5436	34%	0.14	0.15	0.16	0.00
	**WE**					10	707	10,681	6146	88%	0.14	0.14	0.18	0.12

Groups: EN—east-north from the Pelotas River; ES—east-south from the Pelotas River; WE—western distribution. Pop ID—collection site code; BHCB—voucher number at BHCB herbarium (Universidade Federal de Minas Gerais, Belo Horizonte, Brazil); Long—longitude; Lat—latitude; N—individuals number; P—polymorphic sites number; %P—polymorphic sites proportion; π—nucleotide diversity; H_O_—observed heterozygosity; H_E_—expect heterozygosity; *F*_IS_—inbreeding coefficient.

**Figure 2:**
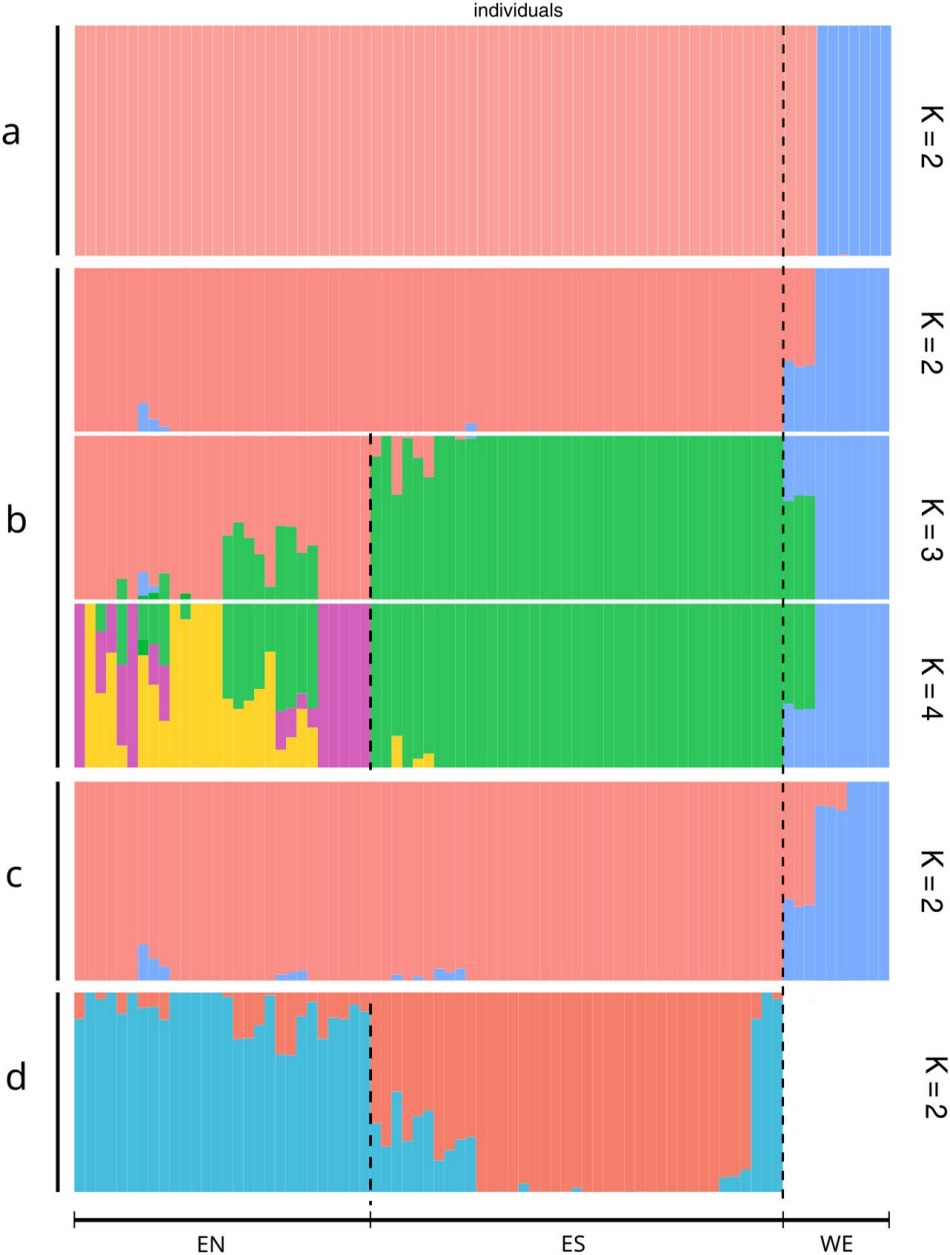
Population genetic structure of *P. altiplana.* (A) DAPC analysis (K = 2) with the first four PCs; (B) fastStructure results (K = 2 to 4). (C) Structure analysis (K = 2); (D) Structure analysis for the E group (K = 2). Dashed lines delimit the groups west (WE) and east, which was divided into east-north (EN) and east-south (ES) subgroups.

The population structure in *P. altiplana* evaluated using DAPC (Fig. 2A) revealed two main groups of populations: one encompassed pop15, pop18, and pop19 [hereafter west (WE) group], distributed in the countryside; the second clustered the eastern (subsequently E group) populations. DAPC analysis retrieved four principal components that resulted in the two main clusters of populations according to the lowest BIC obtained with the *find.clusters* option (Supplementary Information 3: Fig. S1).

**Figure 3:**
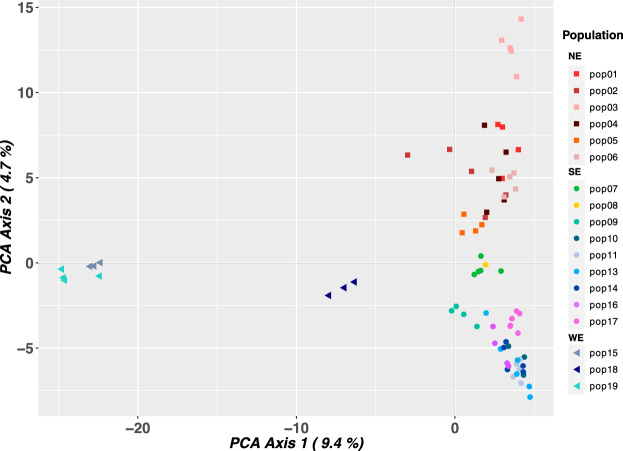
Population genetic diversity. PCA plot showing the distribution of the genetic diversity using the first two PCs. Colored symbols indicate individuals’ distribution, with squares for EN populations, circles for ES group, and triangles for WE populations. Population color follows the legend at right.

The fastStructure (Fig. 2B) yielded equally probable best K values, K = 2, 3, and 4 genetic components. K = 2 indicated that most WE individuals have an exclusive genetic component, whereas some individuals have admixed ancestry; K = 3 differentiated the WE group and divided the E group into two subgroups, one corresponding to populations distributed in the southern Pelotas River riverbank [hereafter east-south (ES)] and the second clustering the populations from the northern Pelotas River margin [hereafter east-north (EN)]. Additionally, with K = 3, some individuals from the EN group were admixed with the ES component, and the same WE admixed individuals in K = 2 now showed admixture with ES. At K = 4, we observed a new genetic component in the EN group. Structure analysis (Fig. 2C; Supplementary Information 4: Fig. S2A) exhibited a best K = 2, where the WE group was differentiated from E populations. Removing WE individuals, Structure analysis (Fig. 2D; Supplementary Information 4: Fig. S2B) exhibited the best K = 2, differentiating the populations of each riverbank with several individuals in each group displaying admixed ancestry. The initial two principal components in PCA (Fig. 3) distinctly segregated individuals, aligning seamlessly with their geographic distribution (Fig. 1), and individuals from each previously identified group exhibited no overlap within the Cartesian plane.

**Figure 4:**
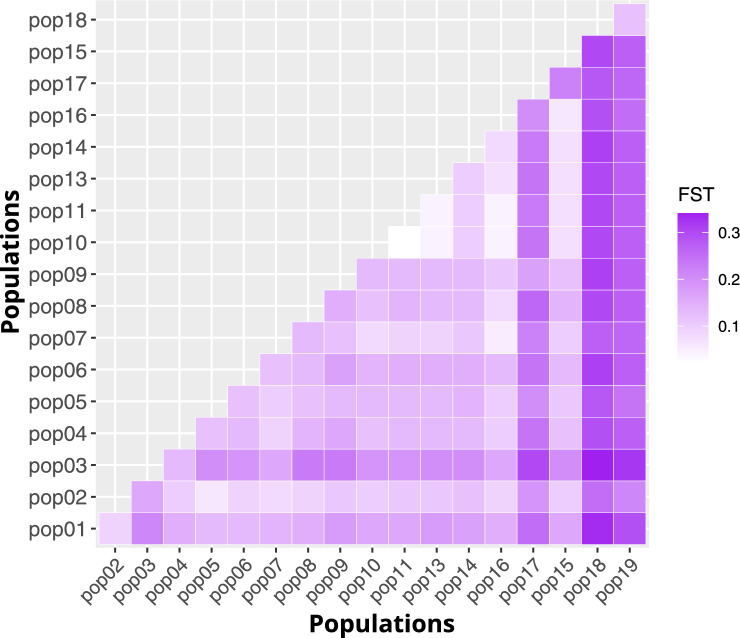
Heat map of pairwise *F*_ST_ values for the populations.

The pairwise *F*_ST_ for collection sites ranged from 0.02 to 0.34 (Fig. 4; Supplementary Information 5: Table S3), and all pairwise comparisons were statistically significant, except for those involving pop8. Compared to the other populations, pop18, and pop19 had the highest *F*_ST_ values (Fig. 4). Regarding the population groups, *F*_ST_ values between WE and both riverbanks’ groups (0.10) than in EN and ES comparison (0.03).

The hierarchical AMOVA revealed that ~ 79% of the total variation was observed within individuals, with low differentiation between groups (WE, EN, and ES; *F*_CT_ = 0.09, p < 0.001) and moderate differentiation between populations within groups (*F*_SC_ = 0.12, *p* < 0.001).

conStruct cross-validation (Supplementary Information 6: Fig. S3) showed that the spatial model was significantly superior to the non-spatial model, indicating isolation by distance between *P. altiplana* populations. For the spatial model, the predictive accuracy was highest at K = 2, with the additional spatial layer 2 contributing very little to the total covariance and the third layer having a significant covariance and deserving to be considered.

### Ecological differentiation between populations

The MRM analysis detected evidence of IBD between populations (R^2^ = 0.19, *p* = 0.03), and the GLMM test revealed that geographical distance better explains the genetic differentiation between all *P. altiplana* populations (Table 2). Further examination of the E subgroups (ES-EN) and WE-ES populations through GLMM revealed that geographical distance combined with elevation was the most explanatory model for genetic differentiation. Pearson’s correlation coefficient indicated that environmental (R^2^ = 0.85; *p* = 0.001) and elevation (R^2^ = 0.70; *p* > 0.001) variables were positively and significantly correlated with geographical distances.

**Table 2:** Generalized linear mixed model (GLMM) tests to evaluate the genetic divergence between *P. altiplana* populations based on three comparisons.

	All			EN vs. ES			EN vs. WE		
	DIC	ΔDIC	DIC weight	DIC	ΔDIC	DIC weight	DIC	ΔDIC	DIC weight
NULL	− 392.68	18.45	0.00	– 324.61	18.47	0.00	– 133.31	5.54	0.03
**GEO**	**− 411.13**	**0.00**	**0.27**	**– 343.08**	**0.00**	**0.80**	– 135.49	3.36	0.09
ENV	− 408.14	2.99	0.06	– 338.02	5.06	0.06	– 134.02	4.83	0.04
ELEV	− 401.23	9.90	0.00	– 331.46	11.62	0.00	– 132.77	6.08	0.02
GEO + ELEV	− 410.37	0.76	0.18	– 133.30	209.79	0.00	– 133.30	5.56	0.03
ENV + ELEV	− 408.37	2.76	0.07	– 131.76	211.32	0.00	– 131.76	7.09	0.01
**GEO + ELEV**	− 409.38	1.74	0.11	– 138.85	204.23	0.00	**– 138.85**	**0.00**	**0.49**
GEO*ENV	− 408.28	2.84	0.07	– 339.43	3.65	0.13	– 131.72	7.13	0.01
GEO + ENV + ELEV	− 409.20	1.93	0.10	– 136.76	206.32	0.00	– 136.76	2.09	0.17
GEO*ENV*ELEV	− 409.71	1.41	0.13	– 135.51	207.58	0.00	– 135.51	3.34	0.09

Considered models: geographic distance (GEO), environmental distance based on soil and climatic variables (ENV), elevation (ELEV). Deviance information criterion (DIC), DIC difference to the best-supported model (ΔDIC), and DIC weight for each model. In bold—best-supported models.

### Evolutionary relationships and migration events

**Figure 5:**
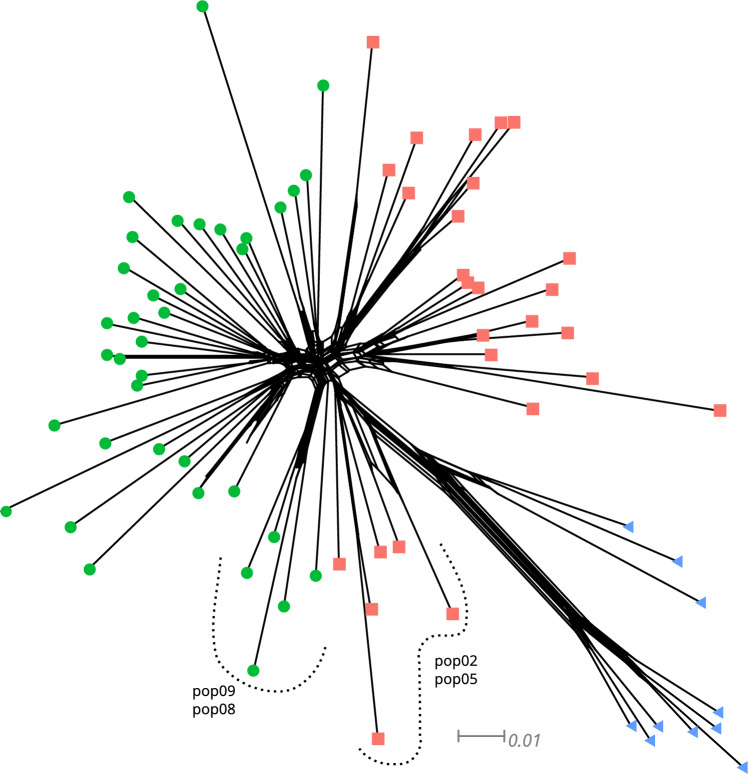
Splitstree phylogenetic network for *P. altiplana* populations and individuals. Green circles indicate ES (east-south), orange squares correspond to EN (east-north), and blue triangles sign WE (west) distributed individuals.

**Figure 6:**
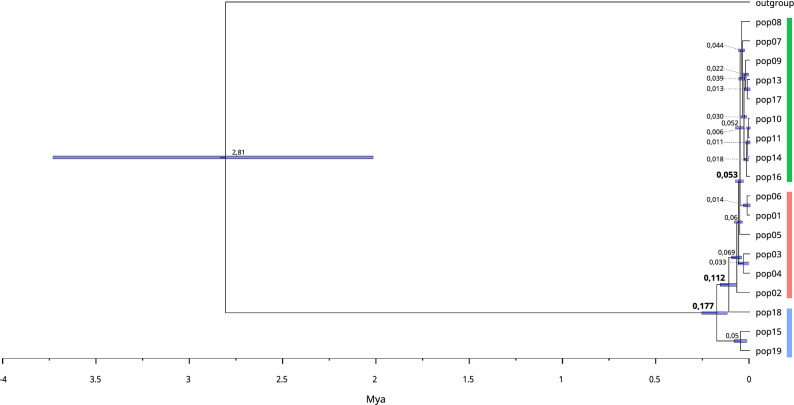
Evolutionary relationships between *P. altiplana* populations obtained using the Bayesian coalescent analysis in SNAPP. Bold values represent the ages of main nodes, and node bars indicate the ages of 95% confidence interval (CI). Colors indicate population groups ES (green), EN (orange), and WE (blue).

The SplitsTree network (Fig. 5A) separated individuals according to their geographical distribution in EN, ES, and WE clades. Groups EN and ES were closer to each other than WE, except some individuals from pop2 and pop5 from EN and pop8 and pop9 from ES were closer to WE populations. This analysis also reflected population structure regarding geographical distribution. The Snapp coalescent analysis (Fig. 6) estimated the divergence time for *P. altiplana* at ~ 2 Mya, with pop15 and pop19 from the WE group as basal to the remaining populations. The estimated divergence time between pop15-pop19 and pop18, also from the WE, was ~ 170 Kya. The division between WE and E occurred at ~ 110 Kya, and the EN and ES populations had divergence time estimated at ~ 50 Kya. The distinction between EN and ES groups is challenging due to a conspicuous pattern of rapid expansion within these population groups, evidenced by their short branches and recent diversification.

The best maximum likelihood tree obtained with Treemix revealed at least four migration events (Supplementary Information 7: Fig. S4A). Although the analysis was inconsistent in depicting the divergence between the populations over the iterations, each iteration at m = 4 inferred a slightly different set of migration edges (Supplementary Information 7: Fig. S4B-F). The populations involved in these migrations were ES (pop07 and pop09), EN (pop02 and pop05), and WE (pop15 and pop19), with gene flow between populations from different groups and inside groups.

**Figure 7:**
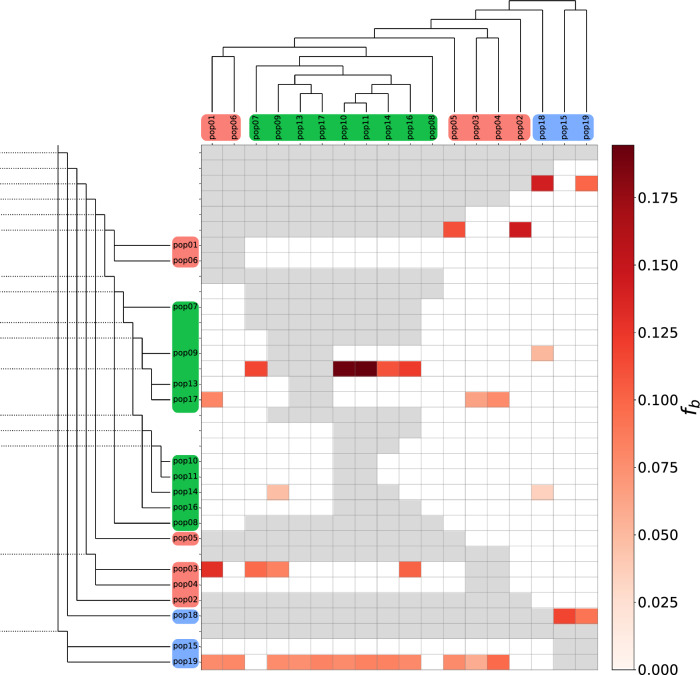
Footprints of migration/gene flow estimated by the *f*-branch statistic. The heat map summarizes the *f*-branch statistics calculated in Dsuite. The SNAPP tree (Fig. 6) was used as a phylogenetic reference tree. Darker colors depict increasing evidence for gene flow between lineages. Dotted lines in the phylogeny represent the ancestral lineage. Rows represent nodes within the tree topology, and columns represent tips. Each cell shows the *f*-branch statistic between a tree node (rows) and each tip (column). Grey cells are empty where comparisons cannot be made. The tree tips are color-coded by the population group: ES (green), EN (orange), and WE (blue).

The *f*-branch statistic indicated multiple introgression or gene flow instances in specific branches and nodes in *P. altiplana* populations (Fig. 7). The *f*-branch test is a heuristic method that aggregates *f*4-admixture ratios throughout the entire tree topology to detect introgression, or, in the case of populations, gene flow throughout internal branches. Gene flow was observed between populations from the same geographic group and between ES and EN branches. Additionally, the WE showed gene flow towards both EN and ES groups, extending to internal branches. Notably, pop19 exhibited the highest frequency of migration towards the other populations. Ancestral gene flow was pronounced in the internal branches linking different populations. The result showed most gene flow inside each riverbank and some events between riverbanks.

**Figure 8:**
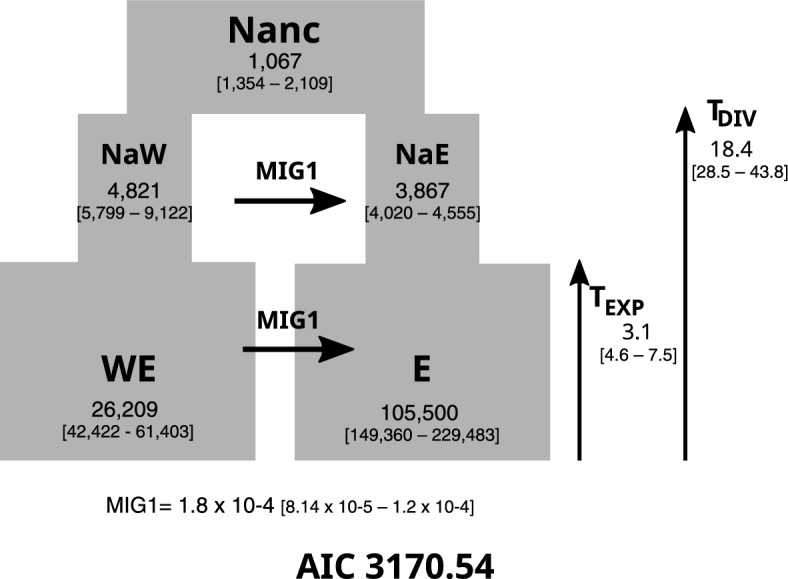
The best scenario obtained in fastSimcoal simulations and estimated values. The 95% confidence interval is assigned below the estimated values. Nanc—ancestral effective population size; NaW and NaE ancestral effective population size for WE and E, respectively, before de expansion. WE and E currently effective population size. T_EXP_—time of expansion (Kya), and T_DIV_—time of divergence expressed in generations (Kya). MIG—unidirectional migration from WE to E.

The first round of fastSimcoal estimations indicated that the best-fit scenario to explain *P. altiplana* demographic history was an expansion event in both the WE and E population main groups (Table 3). Subsequent estimates focusing on gene flow revealed a unidirectional gene flow from WE to E as the best-fit scenario (Fig. 8) (lowest delta likelihood and AIC values—scenario 13) with no likelihood overlap (Supplementary Information 8: Fig. S5). All parameters were presented in Fig. 8 for the best scenario.

**Table 3:** Comparison of demographic models tested with fastSimcoal.

	Scenario	Log10 (L)	**Δ**L	N	AIC	**Δ**AIC
1	Divergence without gene flow	− 49761.14	27281.73	4	229166.52	115160.01
2	Divergence with gene flow	− 27973.343	5493.94	5	128832.01	14825.49
3	Constant expansion in East	− 25971.821	29464.08	6	119616.66	5610.14
4	Expansion in East	− 51943.491	3492.41	5	239218.62	125212.10
5	Constant expansion in West	− 38608.947	16129.54	5	177810.77	63804.26
6	Expansion in West	− 35258.727	12779.32	6	162384.44	48377.93
7	Geral constant expansion	− 55394.147	32914.74	5	255109.47	141102.96
8	Geral expansion	− 25649.949	3170.54	7	118136.38	4129.87
9	Constant gene flow	− 25281.938	2802.53	9	116445.63	2439.12
10	Recent gene flow	− 25642.414	3163.006	11	118109.68	4103.17
11	Early gene flow	− 25640.8	3161.392	11	118102.25	4095.74
12	E to WE gene flow	− 25909.63	3430.222	8	119334.26	5327.74
13	**WE to E gene flow**	**− 24752.725**	**2273.317**	**8**	**114006.51**	**0.00**

*L* likelihood, *AIC* Akaike information criterion.

Bold values indicate the best model.

## Discussion

The phylogeographic history of *Petunia* species has been of interest in recent years, with the genus illustrating several evolutionary processes, including rapid and recent divergence, natural hybridization and introgression, Quaternary refugia, speciation, and genetic diversification in narrowly and widely distributed close species. *Petunia altiplana* is distributed in the subtropical highland grasslands in southern Brazil. The species integrates the *Petunia* short corolla tube clade^16^ and is the only species from the highlands that is widely distributed. There are no taxonomic doubts about the plant’s identity regarding diagnostic morphologic traits considering populations throughout the species distribution. Previous studies revealed that the Pelotas River served as a phylogeographical barrier for the species^17^ despite some shared plastid haplotypes between populations from both riversides^24^. Nuclear microsatellites indicated higher polymorphism-sharing populations from different riverbanks that could be attributed to gene flow, at least in part^24^. We evaluated genetic diversity and population structure based on a large genomic coverture to disentangle the species’ phylogeographical history and clarify the polymorphism-sharing origin.

We obtained high genetic diversity indices, as usually expected for widely distributed species that inhabit heterogeneous environments^27^. The indices were similar to those obtained for other *Petunia* species with wide distribution, such as *P. axillaris* (Lam.) Britton et al. based on SNP variability^7^. Almost all populations (Table 1) showed private alleles, which may be attributed to recent selective pressures, long-term geographical isolation^28^, or even incomplete lineage sorting^29^. The inbreeding coefficient for populations was low, as expected for self-incompatible species such as *P. altiplana*^30^.

Regarding the population structure, based on previous works^17,24^, we expected to observe the Pelotas River as an effective barrier splitting populations from the northern and southern riverbanks. However, most analyses (Fig. 2) indicated that the divergence between EN and ES groups only appears when western populations (WE) are removed. The most marked differentiation was between WE and E populations, which correlates with elevation differences as the WE populations are found in lower elevations. In contrast, EN and ES groups are in the highlands (Fig. 1).

Geographical distribution (Fig. 1) promoted the genetic distinctiveness between populations. PCA’s first two principal components (Fig. 3) consistently separated individuals following their natural geographical arrangement, which indicated a correlation between genetic or environmental factors and geography. Similar patterns were observed for other widely distributed *Petunia* species from countryside lowland grasslands, such *as P. axillaris*^7^, and throughout the Atlantic coastal plain in southernmost Brazil and Uruguay, such as *P. integrifolia* (Hook.) Schinz & Thell^20^.

The conStruct analysis provided compelling evidence for isolation by distance in *P. altiplana*. The spatial model equally supported K = 2 and K = 3. The third genetic layer emerged exclusively among the WE populations (Supplementary Information 6: Fig. S3), reinforcing this group differentiation as seen in other structure analyses. conStruct compares spatial and non-spatial models, and our results pointed out the spatial model in the *P. altiplana* case, underscoring the dominant influence of isolation by distance on population structuring (Supplementary Information 6: Fig. S3B). Such structure suggests a gradual and continuous pattern of genetic differentiation with intermediate populations not sampled or currently extinct.

The GLMM and MRM results confirmed the correlation between geographic distance and genetic diversity and also indicated isolation by distance with elevation as a primary factor (Table 2). Moreover, geographical distance explains the genetic differentiation considering all populations, western and eastern distribution, and between the EN and ES groups from the east. There was a significant correlation between genetic and geographical distances (R^2^ = 0.19, *p* = 0.03) and also between geographical distance and elevation (R^2^ = 0.70; *p* > 0.001). Such correlations indicate that factors such as environmental heterogeneity and geographical barriers could isolate populations by restricting gene flow, as observed for an Atlantic Forest tree (*Bathysa australis* (A.St.-Hil.) K.Schum.)^31^.

Isolation is often found in populations from different elevations^32^, and the environmental conditions can vary significantly over short distances in elevation gradients^33^, which can potentially lead to local adaptation, preventing gene flow, hindering the movement of pollinators and seed dispersers^34^. Small and solitary bees are the *P. altiplana* pollinators (J.R. Stehmann, UFMG, unpublished data), and such bees have low flight autonomy^35^. Moreover, similar to other *Petunia* species, seed dispersal in *P. altiplana* is autochoric^36^, with seeds falling close to the mother plant.

The observed pattern of isolation by distance in *P. altiplana* is consistent with the general trend observed in many widely distributed species as a typical driver of lineages’ differentiation^37^. Some IBD examples exist in *Petunia*^7,20,38^ and other Solanaceae^39^ species from southern South America. All these studies implied a limited gene flow and genetic drift, increasing the population differentiation in these grassland species. However, it is essential to note that geographical distance and elevation alone cannot entirely explain the population structure^37^.

Exploring evolutionary relationships and demographic history among lineages has yielded additional evidence, contributing to understand the current population structure. Notably, the lowland populations from the western distribution (WE population group) are closer to the *P. altiplana* ancestral population (Fig. 5), which aligns with the proposed genus history and the species’ phylogenetic positioning. The *Petunia* genus originated in lowland grasslands within southern South America, from where it dispersed to highland fields during the Pleistocene^16^.

Regarding the eastern-distributed populations (ES and EN groups), the phylogenetic analysis in Splitstree revealed the division between populations from each side of the Pelotas River (Fig. 5). The calibrated tree derived from SNAPP indicated that such divergence between EN and ES groups was recent, occurring in the late Pleistocene (Fig. 6), which reinforced the suggestion that *P. altiplana* originated in lowlands followed by expansion to higher elevations during the Pleistocene. The rapid population expansion in the highlands may not have left enough markers of structuration, contributing to the observed patterns.

The analyses of demographic scenarios (Fig. 7; Table 3) provided robust support for the expansion hypothesis. Among the scenarios assessed with fastSimcoal, the most compelling result portrayed a divergence between the western (WE) and eastern (ES + EN) populations, succeeded by an expansion event. It is worth noting that the estimated diversification time and population sizes might be subject to overestimation because we used the only available mutation rate, which was calculated based on plastid markers. The plastid genome has a lower mutation rate than the nuclear genome and a small effective population size that could bias time estimates^40^.

The highland population expansion dates back to the Pleistocene, estimated at ~ 110 Kya using SNAPP and ~ 18.4 Kya (CI 95%: ~ 28.5 to 43.9 Kya) using fastSimcoal. This temporal framework highlights the significant role of Pleistocene climate changes in shaping the South American biomes, leading to alterations in landscape connectivity and profoundly impacting the current biodiversity distribution^41^. The Pleistocene had an optimal climatic condition for the grasslands’ expansion, while forests contracted during the glacial periods^8^. The forest-adapted species recovered their domains during the interglacial periods, growing from refuges close to the river’s margins^42^, which fragmented grasslands and confined herbs to isolated sky islands^17,19^. Pollen records from the southern Brazilian highlands support this notion by revealing that during the last glacial maximum, the landscape was dominated by grasses and open formations^9^.

Alternatively, *P. altiplana* populations could have migrated to the highlands during interglacial periods to escape from adverse weather conditions. The expansion of the *Araucaria* Forest may have exerted pressure on grassland populations, prompting their movement to higher elevations. Contrary to warmer temperatures during interglacial periods in lowlands, the colder environments in highlands could have been more ecologically favorable to grassland-adapted species. In this scenario, the populations might have sought refuge in the highlands as a strategically better environment^43^.

Regarding gene flow, the Treemix result (Supplementary Information 7: Fig. S4) indicated at least four migration events between population groups. The ABBA-BABA test showed share-derived alleles between the populations, which can be interpreted as ancient gene flow/migration (Fig. 7), especially from pop19 to eastern distributed populations. Such polymorphism sharing^44^ indicates that *P. altiplana* could have been more connected in the past. The best scenario estimated in the fastSimcoal analysis (Fig. 8) pointed to a directional migration more intense from WE to E populations after the group diversification. Our results suggest that the populations were more connected and had gene flow during the expansion to the highlands. The group connection was probably lost with the *Araucaria* Forest’s growth that fragmented the landscape mainly during the Holocene (~ 4 Kya)^9^. The grasslands are now fragmented in isolated high elevations, forming patches of herbaceous plants surrounded by forest^10,45^. Additionally, past and present human activities in the region promote fragmentation and loss of habitat for grassland-adapted species^46^, which has reduced gene flow as patches of wild individuals are isolated by cultivated plants.

As a potential phylogeographical barrier for *P. altiplana,* the Pelotas River appears limited to split ES and EN groups. Whereas no concrete evidence suggests changes in the river’s course and Pelotas River was not implicated in WE and E groups separation, it is crucial to consider documented Pleistocene paleo drainages across South American rivers^47^. Such effects could plausibly have occurred throughout the entire distribution of *P. altiplana*. The paleo drainages serve as an alternative to explain the distribution of sister aquatic lineages^48^, and, in theory, they also can promote landscape fragmentation in grasslands, interrupting population connections through flooding low areas and isolating plant groups, which could prevent the gene flow between them. Similar patterns were observed with *P. axillaris*^7^. The fragmentation could shape microrefugia for grassland-adapted species, which would colonize the region again when flooded areas became reduced. A historical alteration in the Pelotas River course might have played a role in facilitating the expansion of populations to their present distribution. It is conceivable that when the Pelotas River got its current trajectory, combined with the onset of *Araucaria* Forest expansion, the barrier between the populations between the riverside banks began.

## Methods

### Sampling, DNA extraction, and sequencing

We sampled young and healthy leaves from 94 adult individuals distributed in 19 collection sites (hereafter populations) throughout the entire geographical distribution of *P. altiplana* (Fig. 1; Table 1). Plants were identified based on morphological traits following the species’ formal description^24^. The sample size per population adhered to that for non-model species in genomic population analyses^49^ and similar studies involving *Petunia* species^7,20,38^. Vouchers were deposited at the Universidade Federal de Minas Gerais herbarium (BHCB-UFMG) in Belo Horizonte, Brazil.

We powdered the silica-dried leaves with liquid nitrogen to extract genomic DNA following a cetyl-trimethyl ammonium bromide (CTAB; Sigma-Aldrich Chem. Co., St. Louis, USA)-based protocol^50^. We measured DNA concentration with a Qubit Fluorometer (Thermo Fisher Scientific Co., Waltham, USA) and quality with a NanoDrop DN-1000 Spectrophotometer (Thermo Fischer). Finally, we tested for the absence of nucleases using *EcoRI* (NEB—New England BioLabs Inc., Ipswich, USA). DNA samples with 260/280 and 260/230 > 1.80 were considered high quality and used to build genomic libraries.

DNA libraries were processed with DArTseq™ complexity reduction using the combination *PstI-MseI* (NEB) method^51^ and replacing the single adaptor with two ones. Sequencing was performed by bulking equimolar amounts of amplification products from each 96-well microtiter plate sample and using them in a c-Bot’s bridge PCR (Illumina Inc., San Diego, USA), followed by sequencing on the Illumina Hiseq2500 (Illumina).

### Plant guideline statement

Experimental research and field studies on wild plants, including the collection of plant material, complied with relevant institutional, national, and international guidelines and legislation. Appropriate permission has been obtained for collecting plant material following Brazilian law on including genetic material on taxonomic and evolutionary studies under permit number 41530-9/2019.

### Filtering and variant discovering

We used Stacks v2.62^52^ to process the demultiplexed raw data and a de novo SNP calling strategy*.* We examined the quality and specifications of the raw data with FastQC v0.11.7^53^. We removed the barcodes and reads with any adapter contamination or low-quality using default settings in the *process-radtags* Stacks module^54^. FastQC analysis indicated inferior quality in the first four nucleotides, which were removed using Cutadapt v1.16^54^. We used the *denovo map.pl* Stacks module to identify SNPs from reads. We performed the parameter optimization^55^ by running the de novo pipeline multiple times on a subset of 20 individuals, iterating over increasing M = 1–8 and n = 1 to 8 per run. This method seeks the assembly parameters (M and n) that maximize the number of R80 loci in the dataset (the number of polymorphic loci present in at least 80% of samples). The best M = n = 5 parameters were selected, and the de novo mapping was performed.

We used the optimization method for call SNP by deleting samples with high levels of missing data^55^, which increases the overall retention of loci and genotypes after filtering and reducing biases due to missing data (in this process, the pop12 was removed). The final dataset encompassed 77 individuals from 18 collection sites. We filtered the missing data using the *population* Stacks module, retaining only loci in at least 80% of the individuals across populations (R = 0.8). We set the minor allele frequency (MAF) cutoff at 0.04. We used only the first SNP of each read (—*write-single-snp*), preventing linkage disequilibrium. We identified the outlier SNPs using PCAdapt v3.5.1^56^. The final *vcf* file with only neutral SNPs was converted to different formats using PGDSpider v2.1.1.2^57^ and dartR v2.05^58^ R package to perform further analyses.

### Genetic diversity and population structure

We estimated nucleotide diversity (π), allelic richness (AR), private alleles (PA), observed (H_O_) and expected (H_E_) heterozygosities, mean inbreeding coefficient (*F*_IS_) per population, and fixation index (*F*_ST_) with *population* Stacks module.

We ran a multivariate discriminant analysis of principal components (DAPC)^59^ to evaluate population structure and individuals’ ancestry using the *find.cluster* and *optim.a.score* options in adegenet v2.1.3^60^ R package. Similarly, we used fastStructure v1.0^61^ and Structure v2.3.4^62^ approaches. fastStructure employs a variational Bayesian framework to compute posterior distributions and identifies dataset clusters. To determine the optimal number of groups, we ran fastStructure from K = 1–18 using the logistic prior and the *chooseK* function. With Structure, we investigated the number of population clusters and potential admixture between populations using MCMC. Hierarchical analyses were performed for ten runs per K, up to a maximum of six, and the admixture model was used with a burn-in of 10,000 steps followed by 100,000 steps. We ran fastStructure and Structure analyses with the Structure_threader software^63^, summarized results using Structure Harvester^64^, and evaluated the likely number of populations based on the inspection of likelihood plots and the Evanno method^64^. We used pophelper^65^ to plot the fastStructure and Structure graphs. We also ran Structure excluding some populations using the same parameters above and K = 1–5 due to preliminary results. We ran a principal components analysis (PCA) to evaluate the structuring between populations and estimate admixture using dartR^58^.

Finally, we ran a hierarchical Analysis of Molecular Variance (AMOVA)^66^ using Arlequin v3.5.2.2^67^, considering individuals, all populations, and groups of populations following structure results.

To incorporate geographical information along with SNPs, we used conStruct^68^. We employed the cross-validation method, conducting analyses with eight replicates, K = 1–5, 10,000 MCMC iterations sampled every 1000, and a 0.5 training proportion. Subsequently, analyses with K = 2–5 were conducted, with ten replicates, using one chain running, 100,000 MCMC iterations sampled every 1000 iterations, and a spatial model.

### Genetic differences as a function of geography and environment

We used a generalized linear mixed model (GLMM) to determine if geographical distance, climate and soil variables, and elevation could explain the population differentiation. Based on preliminary results, we subdivided the GLMM analysis into the best number groups based on the structure analysis (DAPC, fastStructure, and Structure results), comparing groups of populations to determine whether the same variables can equally explain the divergence between groups. As response variables, we created a matrix of pairwise *F*_ST_ distances {*F*_ST_/(1 − *F*_ST_)^69^}. We evaluated three distinct matrices as potential predictor variables: (1) GEO, which included the pairwise geographical distances between populations; (2) ENV, based on the Euclidian distances along the first three axes of a PCA for 35 climate variables extracted from CliMond^70^ and nine soil variables obtained from SoilGrids^71^ with a resolution of 30′′ (c. 1 km^2^; Supplementary Information 9: Table S4); and (3) ELEV, which included the pairwise elevation distance between populations. We extracted elevation data from the coordinates of the collection sites. We executed GLMM within the MCMC_GLMM_^72^ R package and previously published scripts^73^ adapted to the current data. We evaluated ten models derived from the combination of the GEO, ENV, and ELEV variables and a null model with no predictors. We compared the models using the deviance information criterion (DIC) and associated DIC differences to determine which model better explained the genetic divergence between populations. We ran MCMC_GLMM_ with standard priors and a burn-in of 500,000 iterations, followed by two million iterations with a thinning interval of 750 steps. We confirmed the chain convergence of MCMC_GLMM_ using the CODA^74^ R package to examine trace plots. We determined how environmental variables correlated to geography using Pearson’s correlation coefficient implemented in the HMISC v4.4-1^75^ R package. We also used the pairwise F_ST_ and geographical distances between populations to test for IBD through multiple regression on matrices (MRM) in the ecodist^76^ R package.

### Evolutionary relationships and gene flow

To investigate evolutionary relationships between populations or groups of populations, we construct a relationship network using the NeighborNet method in Splitstree v4.16^77^. Additionally, we employed a coalescent framework using the SNAPP v1.3^78^ implemented in Beast2 v2.4^79^ to infer the evolutionary relationships between populations based on SNP data. SNAPP calculates the probability of the species tree without gene trees, mathematically integrating all possible gene trees. We used the previously described approach^80^, which tweaks the SNAPP’s settings to include a strict clock model that can be time-calibrated based on the fossil record or information from other phylogenies. We employed secondary calibration obtained in previously published studies to calibrate the molecular clock^18^. The calibration was based on the divergence time between the *Petunia* short and long corolla tube clades (2.85 million years ago). We used *P. axillaris,* which belongs to the long corolla tube clade, as an outgroup. A standard deviation of 0.16 in real space was applied in a normal distribution using BEAUTi as part of the BEAST package^79^. For computational efficiency, we subsampled populations, including one representative per each (overall 18 individuals) + one individual as an outgroup. We limited the dataset to 1000 randomly selected SNPs and set the chain length at 100,000 MCMC iterations. We assessed runs using Tracer v1.6^81^ to examine convergence (ESS > 200) and tree topologies, and we visualized node heights using Densitree^82^ and FigTree v1.4.4 (https://github.com/rambaut/figtree/).

To generate a phylogenetic tree that accommodates admixture, we used the TreeMix v1.13 software^83^. We reduced the number of populations to improve the resolution of the analysis by clustering closely related populations, as observed in the SNAPP tree. Additionally, we removed pop08 due it had only one sampled individual, resulting in ten populations: EN1 (pop01 + pop06); EN2 (pop02); EN3 (pop03 + pop04); EN4 (pop5); ES5 (pop7); ES6 (pop09 + pop13 + pop17); ES7 (pop10 + pop11 + pop14 + pop16); ES8 (pop15 + pop19); and ES9 (pop18). The optimal number of migration edges on a population tree containing one to ten edges was estimated using the OptM^84^ R package.

To test the potential migrants/gene flow between the populations, we performed the ABBA-BABA using Dsuite v0.3^85^. ABBA–BABA statistic is based on the number of ancestral (A) and derived (B) alleles in a four taxa phylogeny as (((P1,P2),P3),O), where O is the outgroup and P1, P2, and P3 are the target populations. If targets did not hybridize, the number of shared alleles between P1 and P3 (BABA) or P2 and P3 (ABBA) should be equivalent. In contrast, excessive sharing indicates hybridization between the populations. In ABBA-BABA analysis, we also used the SNAPP tree as a reference and *P. axillaris* as an outgroup. The analysis considers incomplete lineage sorting as the null hypothesis^86^. The *D* and *f*_4_-ratio statistics were calculated using the *Dtrios* function in Dsuite with default parameters. For better interpretation, the results from *Dtrios* were further processed using the Fbranch software and associated plotting utilities for the *f*-branch statistic.

### Demographic modeling

To explore alternative demographic models for *P. altiplana* populations, we estimated demographic scenarios in fastSimcoal v2.6^87^. We tested 13 evolutionary scenarios using a hierarchical approach to identify which scenario better explains the demographic history of *P. altiplana.* For the division of the populations, we followed the number of groups of DAPC, Structure, and phylogenetic relationships. We started with an exploration of multiple scenarios of expansion, differentiating between the population groups: (1) divergence without gene flow; (2) divergence with gene flow; (3) constant expansion in the first group; (4) an event of expansion in the first group; (5) constant expansion in the second group; (6) an event of expansion second group; (7) constant expansion on both groups; and (8) an event of expansion in both groups. Finally, we assessed gene flow in the best-fitting model, considering (9) constant gene flow; (10) recent gene flow; (11) ancient gene flow; (12) unidirectional gene flow from the first to second group; and (13) unidirectional gene flow from second to first group (Supplementary Information 10: Fig. S6).

The site frequency spectra were estimated with easySFS software (https://github.com/isaacovercast/easySFS) using the VCF file without filtering by *–min-maf,* and samples were projected downward to maximize the number of loci without missing data *vs*. the number of retained individuals. Groups of populations followed previous results for population structure, with a projection of 108 and 14 haploid samples for each group, respectively. We used an overall substitution rate of 2.8 × 10^−9^ per site/generation, as reported for *Petunia*^17^. For each tested demographic model, we performed 100 independent runs using 100,000 simulations, 40 expectation–maximization cycles, and a broad search range for parameters (Supplementary Information 11: Table S5) to determine the run with the best parameter estimates and maximum likelihood. The 13 scenarios were compared using Akaike’s information criterion (AIC)^88^ to select the best-fitting demographic model. We ran each model with its best parameters 100 times and compared the likelihood distributions to check whether the models were significantly different or just stochastic results. We calculated the 95% confidence intervals for each estimated parameter using 100 non-parametric bootstrap SFS.

### Supplementary Information

Supplementary Information.

## Data Availability

The original unfiltered VCF dataset is available at https://figshare.com/s/57a3c19a8363498bf168, bioproject https://www.ncbi.nlm.nih.gov/bioproject/PRJNA931913. Raw reads are available at https://www.ncbi.nlm.nih.gov/sra/ under codes SRR23348487 to SRR23348590.
