# Supplementary material for: The phylogeographic journey of a plant species from lowland to highlands during the Pleistocene
Source: Sci Rep. 2024 Feb 15;14:3825. doi: 10.1038/s41598-024-53414-4 (PMC10869790; doi:10.1038/s41598-024-53414-4)
Supplement: Supplementary file 1 — Supplementary Information. [file 41598_2024_53414_MOESM1_ESM.pdf]

Supplementary Information

The phylogeographic journey of a plant species from lowland to highlands during the Pleistocene

LUANA SOUSA SOARES, LORETA B. FREITAS*

Department of Genetics, Universidade Federal do Rio Grande do Sul, Porto Alegre, Brazil;

**Corresponding author*: Loreta B. Freitas. Department of Genetics, Universidade Federal do Rio Grande do Sul, Porto Alegre, Brazil. PoBox 15053; 91501-970. E-mail: loreta.freitas@ufrgs.br.

Running title: *Petunia altiplana* evolutionary history

Supplementary Information 1: Table S1. Information regarding the number of barcodes, reads generated per sample on DART-seq analysis, and quality filter based on STACKS *process_radtags* for *Petunia altiplana*.

				Reads			
**pop**	**Individual**	**Sample ID**	**Barcode**	**Total**	**Adapter sequences**	**Low quality**	**Processed**
pop01	Palti22	2062164	GTCTACGTCTTCAAT	1,494,776	539,824	290	954,662
	Palti23	2062165	TATCCTAACTTGCAG	1,608,830	675,345	298	933,187
	Palti24	2062166	CGCCTGTGAGGTTAT	2,151,254	853,141	370	1,297,743
	Palti25	2062167	CCGCTCGGTATTGCA	2,173,840	943,199	403	1,230,238
pop02	Palti26	2062168	ATACCTATAGATGCA	2,173,612	935,930	385	1,237,297
	Palti27	2062169	CTGAGCACAGTGCAG	1,761,986	759,136	321	1,002,529
	Palti28	2062170	TTATACTTGCAATGC	1,549,700	607,968	284	941,448
	Palti53	2062171	GAATGAGTGCCGATG	1,707,138	641,322	315	1,065,501
	Palti71	2062172	CGCAATGAGGATGCA	1,613,844	685,098	286	928,460
pop03	Palti88	2062173	ATTGAACGCAATGCA	1,606,363	701,562	325	904,476
	Palti90	2062174	GCGAAGGAATAGATG	2,200,728	874,312	431	1,325,985
	Palti94	2062175	ACGATAATCTATTGC	2,229,221	869,589	413	1,359,219
	Palti100	2062176	GAAGTATGCGGTCGT	1,925,787	705,545	330	1,219,912
	Palti102	2062177	TGTTCAATATTGCAG	1,918,502	783,020	306	1,135,176
pop04	Palti36	2062178	GCCTTCAGTGTGCAG	1,521,224	568,007	254	952,963
	Palti132	2062179	TTGCACACGTTGCAG	1,493,440	580,373	279	912,788
	Palti136	2062180	AATCCATGGTCGTGC	1,550,060	620,145	285	929,630
	Palti139	2062181	CAAGTGCAGTGGAAT	1,646,145	674,198	295	971,652
	Palti146	2062182	ACACGGTGAGTGCAG	2,070,226	851,726	370	1,218,130
pop05	Palti277	2062183	ATTGACACCTGATAT	2,165,801	813,822	378	1,351,601
	Palti292	2062185	CATCAAGAATGATTG	1,717,152	642,556	305	1,074,291
	Palti293	2062186	GTCTATGGAGTGCAG	1,435,756	512,134	228	923,394
	Palti296	2062187	TGAACGCATCCGATT	1,727,978	706,821	280	1,020,877
pop06	Palti320	2062150	CCATGTTCTCACTTG	1,892,062	750,172	365	1,141,525
	Palti321	2062151	ACTGGAGACTTGCAG	1,949,009	748,007	369	1,200,633
	Palti323	2062152	CTTAGTAACTCCAAT	1,566,527	576,792	294	989,441
	Palti324	2062153	TCTCTGAATAACGTG	1,623,847	626,641	282	996,924
	Palti325	2062154	GCGGTCAATATGCAG	1,744,975	679,491	328	1,065,156
pop07	Palti148	2062188	GCGAGGAGTATGCAG	1,511,202	596,891	295	914,016
	Palti150	2062189	TGGTCGTGAACCTAT	1,444,958	575,282	293	869,383
	Palti154	2062191	CATATCGGACTCTAT	2,086,484	828,368	391	1,257,725
	Palti149	2062192	TATCATCTCTATGCA	1,880,107	763,863	325	1,115,919
	Palti153	2070207	GACAGAGAGATGCAG	1,448,507	808,310	240	639,957
pop08	Palti238	2062196	CATAAGCCATTGCAG	1,585,698	624,791	297	960,610
pop09	Palti297	2062198	GTCTGTTCGCCATGC	1,686,569	603,749	291	1,082,529
	Palti305	2062199	GAGGTCGCTAATGCA	1,978,938	735,650	333	1,242,955

	Palti302	2062200	ATATGCAACTATGTG	2,350,379	981,183	446	1,368,750
	Palti306	2062202	TCCGATCCAGAATGC	1,684,194	654,870	296	1,029,028
pop10	Palti214	2062205	ACTTGGCTTCTGCTT	1,643,100	734,384	316	908,400
	Palti190	2062206	CGGCTTCCTTGTTGC	2,088,945	841,367	415	1,247,163
	Palti193	2062207	GCCTGTCAATGGATG	2,454,436	1009,090	433	1,444,913
pop11	Palti7J	2062208	TGTTGTAGCAGCGGT	2,023,906	730,624	365	1,292,917
	Palti8J	2062209	TATATACTGTCAGTG	1,900,983	748,218	350	1,152,415
	Palti9J	2062210	TATAGAGTCGCGTGC	1,444,642	523,370	246	921,026
	Palti13	2062211	GATCACCTAAGTGCA	2,001,022	911,649	334	1,089,039
	Palti16	2062212	GCACGACCGAATCTT	1,866,653	813,948	295	1,052,410
	Palti18	2062213	TTAGTTCTGGTCAAT	1,428,287	596,312	249	831,726
pop13	Palti46	2062217	GACGTAGTAGGTGCA	2,227,037	960,329	429	1,266,279
	Palti49	2062218	CTACTGTAGGATGCA	2,150,076	963,799	376	1,185,901
	Palti50	2062219	CTGATGAGGTTCGTG	2,060,371	1,006,811	348	1,053,212
	Palti51	2062220	CCAGACCGTGTGCAG	1,738,027	742,364	345	995,318
	Palti47	2062221	TCCAAGTGCAAGTGC	1,949,919	1,018,975	344	930,600
pop14	Palti232	2062222	TAGCACGCATACTTG	2,375,042	895,033	446	1,479,563
	Palti228	2062223	AGGTGCTGCCTTATG	2,320,800	886,199	448	1,434,153
	Palti164	2062224	GATTCAAGTGATGCA	2,319,531	896,632	422	1,422,477
	Palti170	2062225	GCTGGAACTCGGTGC	2,045,918	786,398	334	1,259,186
	Palti173	2062226	CCGCGATACTGAGTG	2,092,494	870,430	399	1,221,665
pop15	Palti160	2062227	GACTGATCCAATGCA	1,889,036	769,198	335	1,119,503
	Palti161	2062228	ATATCAGGAGCAGTG	1,588,468	627,465	299	960,704
	Palti162	2062229	CCAGAACAACTTGCA	1,759,341	746,418	322	1,012,601
pop16	Palti253	2062230	CTTCGCTTATTATTG	1,752,960	660,932	346	1,091,682
	Palti256	2062231	TGGATTATTGCTGTG	2,685,495	1,094,471	476	1,590,548
	Palti257	2062232	TGAGCGATGGATTGC	2,196,651	919,543	400	1,276,708
	Palti260	2062233	GAACTCTAAGCGTGC	2,249,408	842,102	391	1,406,915
pop17	Palti261	2062234	TCTTGGTCTCCATGC	1,954,566	789,728	345	1,164,493
	Palti263	2062235	ACTTGCATAACTTGC	2,120,365	870,314	371	1,249,680
	Palti265	2062236	AAGACATAAGATTGC	1,922,573	796,713	342	1,125,518
	Palti267	2062237	GTCACCAACTATAAT	1,648,852	643,303	290	1,005,259
	Palti272	2062238	AACACACCTGTGTGC	3,342,929	1,343,528	608	1,998,793
	Palti275	2062239	TAGCCTATCGGCAGT	2,894,168	1,234,703	536	1,658,929
pop18	Palti309	2062240	ACGAGTGTAAGTGCA	1,632,825	722,461	306	910,058
	Palti313	2062241	TTGCAGCCACCGAGT	2,340,910	922,568	431	1,417,911
	Palti314	2062242	TAGATGCGTGTGCAG	2,043,055	900,565	342	1,142,148
pop19	Palti330	2062155	AGCATTGTTATTGCA	1,660,858	665,751	304	994,803
	Palti332	2062156	AAGATGTAGATGCAG	1,578,501	631,103	282	947,116
	Palti336	2062157	TAGACGTCCTGTGCA	1,570,394	655,979	288	914,127
	Palti337	2062158	CAGCCGAATAATGCA	2,271,090	970,266	422	1,300,402
Total				147,110,453	59,841,876	26,606	87,241,971

Supplementary Information 2: Table S2. Variant calling obtained using STACKS and filtered SNPs.

Pop	Private	Sites	Polymorphic sites	SNPs	Neutral SNPs
pop01	8,434	8,282,348	55,689	306,677	10,680
pop02	17,299	7,306,466	64,954	307,023	10,679
pop03	9,955	6,705,885	52,809	305,841	10,674
pop04	10,760	7,113,277	57,296	307,591	10,683
pop05	12,095	11,117,499	56,552	311,789	10,669
pop06	13,121	8,335,645	75,018	330,395	10,683
pop07	12,221	8,834,605	55,947	304,129	10,677
pop08	6,072	5,345,993	24,471	242,426	9,193
pop09	11,056	7,566,430	54,908	307,156	10,671
pop10	9,082	8,636,859	50,137	297,016	10,671
pop11	16,461	8,509,856	73,065	321,259	10,683
pop13	12,431	9,866,795	60,877	313,125	10,683
pop14	14,077	8,822,366	64,653	318,973	10,680
pop15	12,920	5,871,585	42,796	269,503	10,613
pop16	10,093	6,906,486	48,457	294,311	10,678
pop17	15,800	8,426,988	75,667	325,175	10,682
pop18	13,021	7,953,774	42,671	286,088	10,562
pop19	19,987	8,450,423	57,443	294,110	10,644

(A)

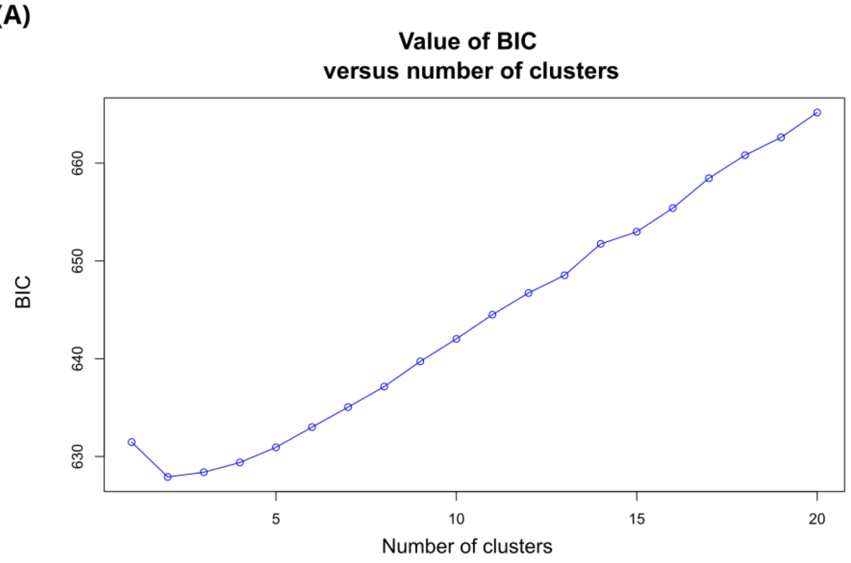

(B)

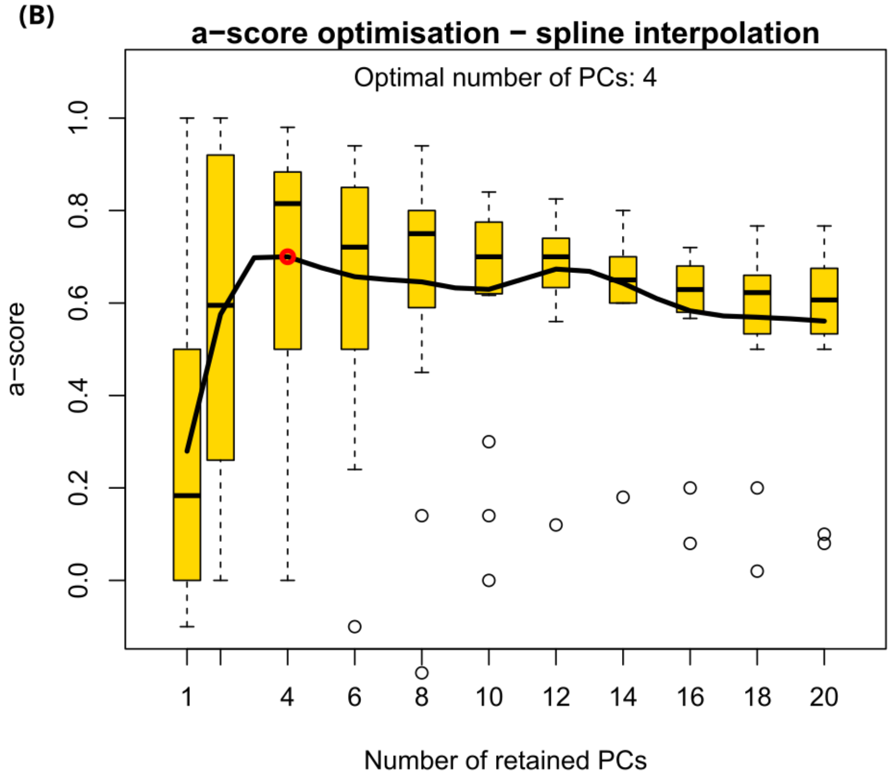

Supplementary Information 3: Figure S1. DAPC analysis results. (A) BIC score of the *find.cluster* analysis (B) a-score optimization of the optimal number of PCs.

A)

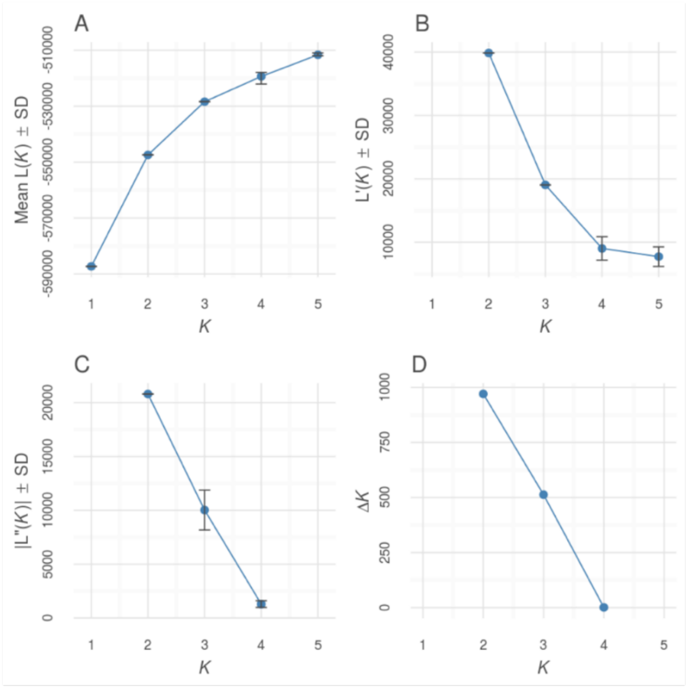

B)

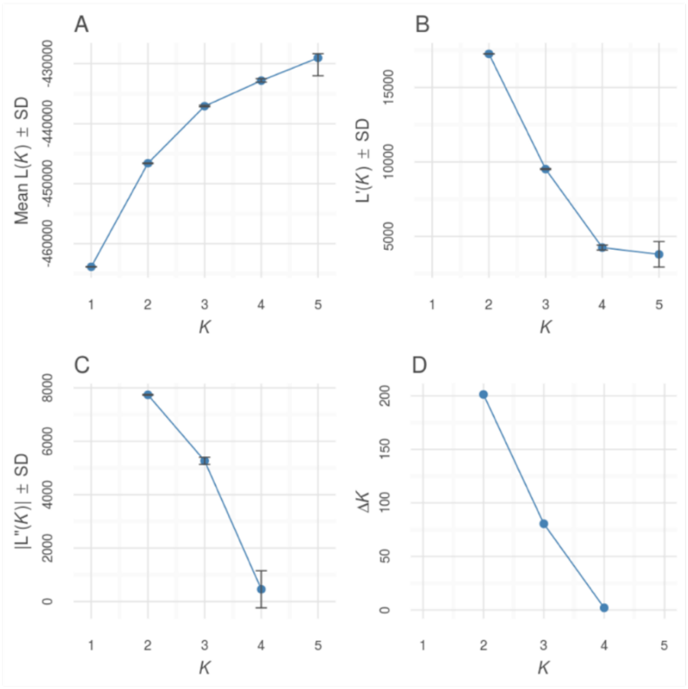

Supplementary Information 4: Figure S2. Evanno's test for Structure considering all individuals (A) and only E individuals (B); best K = 2 for both analyses. Each test shows: A - estimated log probability of data of runs over increasing values of K; B - first derivative; C - second derivative; and D - ΔK over values of K.

Supplementary Information 5: Table S3. Pairwise F_{ST} values for *P. altiplana* populations: EN- pink; ES – green; WE - blue.

	pop01	pop02	pop03	pop04	pop05	pop06	pop07	pop08	pop09	pop10	pop11	pop13	pop14	pop15	pop16	pop17	pop18	pop19
pop01																		
pop02	0.09																	
pop03	0.27	0.19																
pop04	0.17	0.11	0.16															
pop05	0.15	0.07	0.25	0.14														
pop06	0.15	0.1	0.24	0.15	0.14													
pop07	0.17	0.09	0.2	0.1	0.11	0.13												
pop08	0.17	0.1	0.3	0.16	0.13	0.15	0.15											
pop09	0.22	0.13	0.3	0.2	0.15	0.21	0.14	0.18										
pop10	0.19	0.11	0.23	0.13	0.15	0.16	0.09	0.14	0.15									
pop11	0.2	0.13	0.24	0.14	0.15	0.17	0.09	0.17	0.16	0.02								
pop13	0.21	0.12	0.25	0.15	0.15	0.18	0.1	0.15	0.15	0.04	0.04							
pop14	0.21	0.14	0.25	0.15	0.16	0.18	0.13	0.15	0.14	0.11	0.11	0.11						
pop15	0.2	0.11	0.25	0.13	0.13	0.15	0.11	0.16	0.13	0.07	0.07	0.08	0.07					
pop16	0.18	0.1	0.19	0.11	0.11	0.15	0.05	0.09	0.12	0.04	0.04	0.07	0.09	0.06				
pop17	0.34	0.23	0.44	0.31	0.25	0.31	0.28	0.36	0.2	0.32	0.3	0.31	0.29	0.29	0.25			
pop18	0.49	0.34	0.53	0.42	0.39	0.45	0.37	0.43	0.45	0.43	0.42	0.43	0.44	0.42	0.4	0.38		
pop19	0.41	0.26	0.47	0.36	0.31	0.38	0.35	0.36	0.37	0.36	0.37	0.37	0.36	0.36	0.33	0.35	0.13	

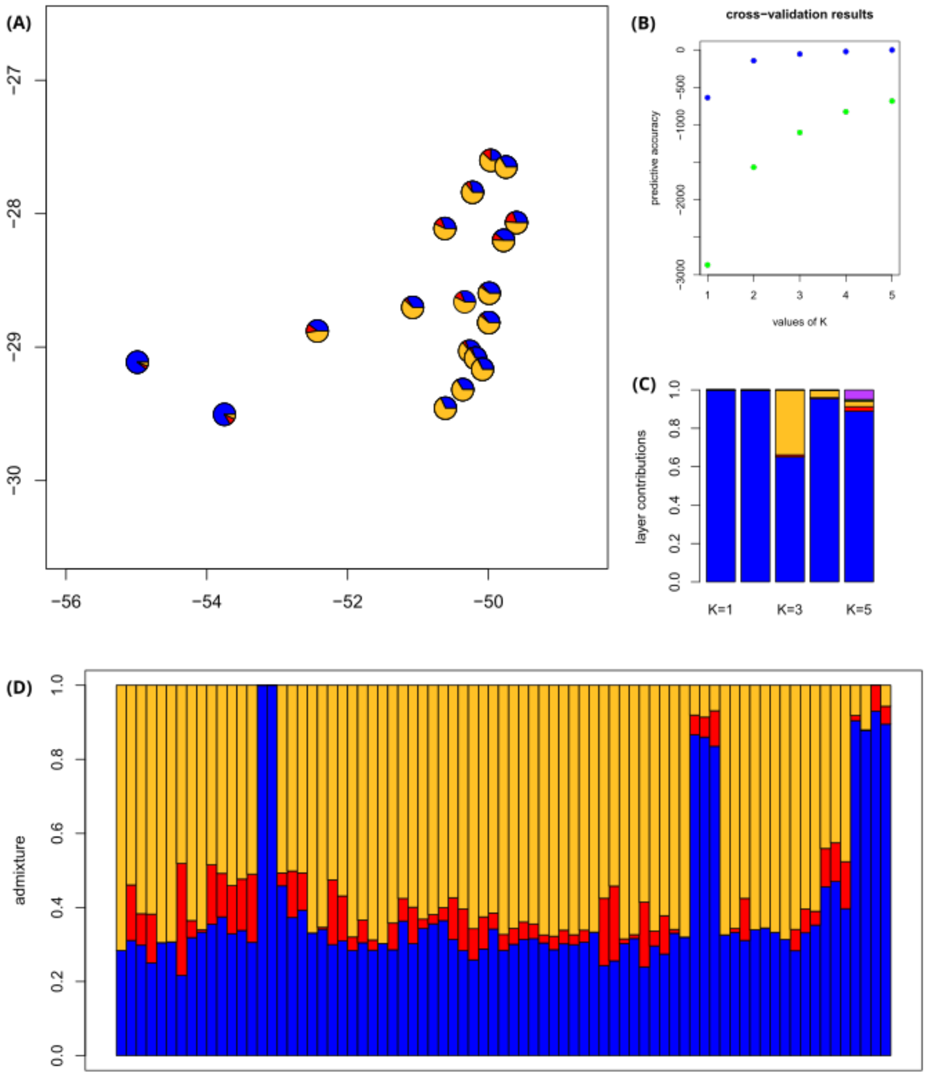

Supplementary Information 6: Figure S3. conStruct results. (A) Pie map with the layers' contribution of the populations; (B) cross-validation of spatial (blue) and non-spatial (green) models; (C) layer contributions K = 1 to 5; (D) structure plot for k = 3 layers of the individuals.

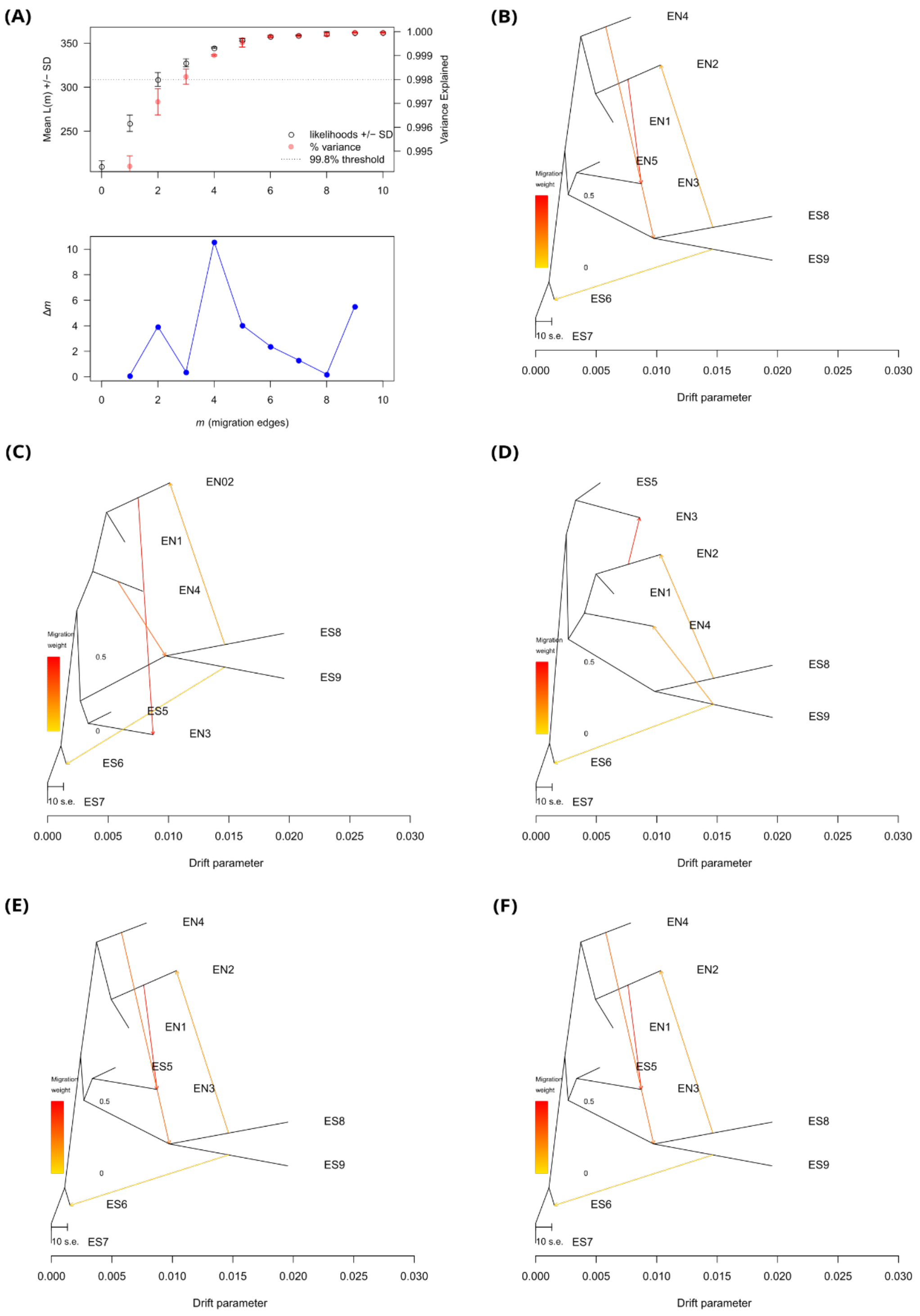

Supplementary Information 7: Figure S4. Treemix analyses. (A) Selection of the most likely number of migration edges by plotting deltaM (OptM package). Distribution of log-likelihood and variance explained of Treemix models with 0 - 10 migration edges. (B – F) The Treemix graph with the optimal number of migration edges identified by OptM m = 4 in five different iterations. EN1 (pop01 + pop06); EN2 (pop02); EN3 (pop03 + pop04); EN4 (pop5); ES5 (pop7); ES6 (pop09 + pop13 + pop17); ES7 (pop10 + pop11 + pop14 + pop16); ES8 (pop15 + pop19); and ES9 (pop18).

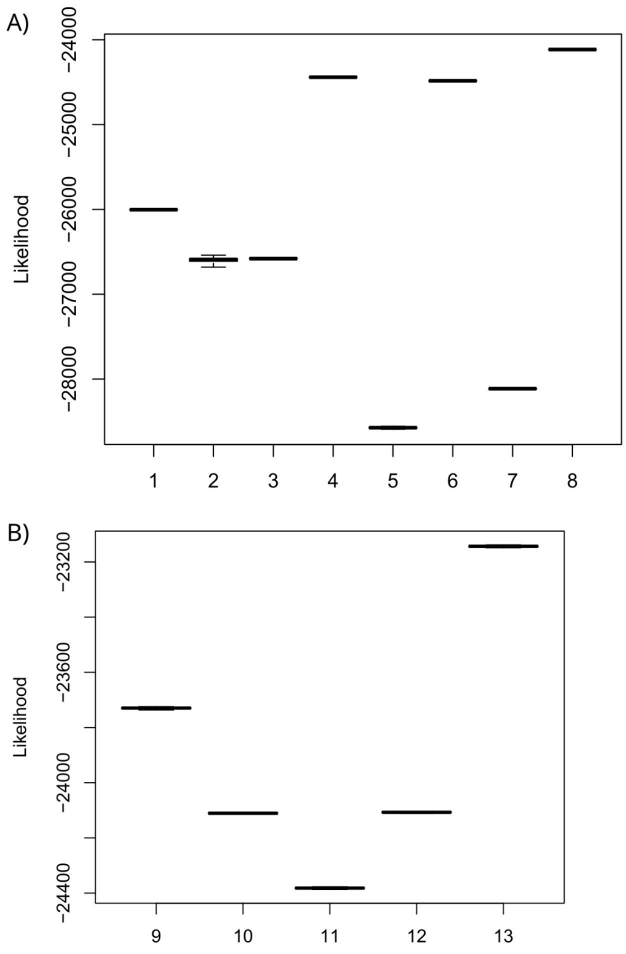

Supplementary Information 8: Figure S5. Likelihood comparisons between the fastSimcoal demographic scenarios. (A) scenarios of divergence and expansion; (B) scenarios testing gene flow. Scenarios: 1 - Divergence without geneflow; 2 - Divergence with gene flow; 3 - Constant expansion in E group; 4 - Expansion in E group; 5 - Constant expansion in WE group; 6 - Expansion in WE group; 7 - Overall constant expansion; 8 - Overall expansion; 9 - Constant gene flow; 10 - Recent gene flow; 11- Early gene flow; 12 - Gene flow from E to WE; 13 – Gene flow from WE to E. Nanc – ancestral effective population size; NaW and NaE ancestral effective population size for WE and E, respectively, before expansion. WE and E currently effective population size. T_{EXP} – time of expansion in generations, and T_{DIV} – time of divergence in generations. MIG – unidirectional migration from WE to E.

Supplementary Information 9: Table S4. Climate and soil variables and their respective references used in GLMM analyses.

Climate	Soil
Annual mean temperature (°C)	Bulk density (fine earth) in Kg / cubic-meter at depth 1
Mean diurnal temperature range (mean (period max-min)) (°C)	Cation exchange capacity of soil in cmolc/kg at depth 1m
Isothermality	Clay content (0-2 micro meter) mass fraction in % at depth 1m
Temperature seasonality (C of V)	Coarse fragments volumetric in % at depth 1m
Max temperature of warmest week (°C)	Soil Organic Carbon Stock in tons per ha for depth interval 0.6 – 1m
Min temperature of coldest week (°C)	Soil organic carbon content (fine earth fraction) in g per kg at depth 1m
Temperature annual range (°C)	Soil pH x 10 in H2O at depth 1m
Mean temperature of wettest quarter (°C)	Soil pH x 10 in KCl at depth 1m
Mean temperature of driest quarter (°C)	Silt content (2-50 micro meter) mass fraction in % at depth 1m
Mean temperature of warmest quarter (°C)	
Mean temperature of coldest quarter (°C)	
Annual precipitation (mm)	
Precipitation of wettest week (mm)	
Precipitation of driest week (mm)	
Precipitation seasonality (C of V)	
Precipitation of wettest quarter (mm)	
Precipitation of driest quarter (mm)	
Precipitation of warmest quarter (mm)	
Precipitation of coldest quarter (mm)	
Annual mean radiation (W m-2)	
Highest weekly radiation (W m-2)	
Lowest weekly radiation (W m-2	
Radiation seasonality (C of V)	
Radiation of wettest quarter (W m-2)	
Radiation of driest quarter (W m-2)	
Radiation of warmest quarter (W m-2)	
Radiation of coldest quarter (W m-2)	
Annual mean moisture index	
Highest weekly moisture index	
Lowest weekly moisture index	
Moisture index seasonality (C of V)	
Mean moisture index of wettest quarter	

Mean moisture index of driest quarter
Mean moisture index of warmest quarter
Mean moisture index of coldest quarter
Mean Wind Speed (m s-1)

Soil variables were obtained from:

Hengl, T. *et al.* SoilGrids1km—global soil information based on automated mapping. *PLoS ONE* **9**, e105992 (2014).

Hengl, T. *et al.* SoilGrids250m: global gridded soil information based on Machine Learning. *PLoS ONE* **12**, e0169748 (2017).

Soil variables were modeled for 2016 based on land covers from 2010.

Climate variables were obtained from:

Hutchinson, M., Xu, T., Houlder, D., Nix, H., McMahon, J. ANUCLIM 6.0 User's Guide. Australian National University, Fenner School of Environment and Society (2009).

Climate variables were obtained as annual mean values from 1970 to 2000.

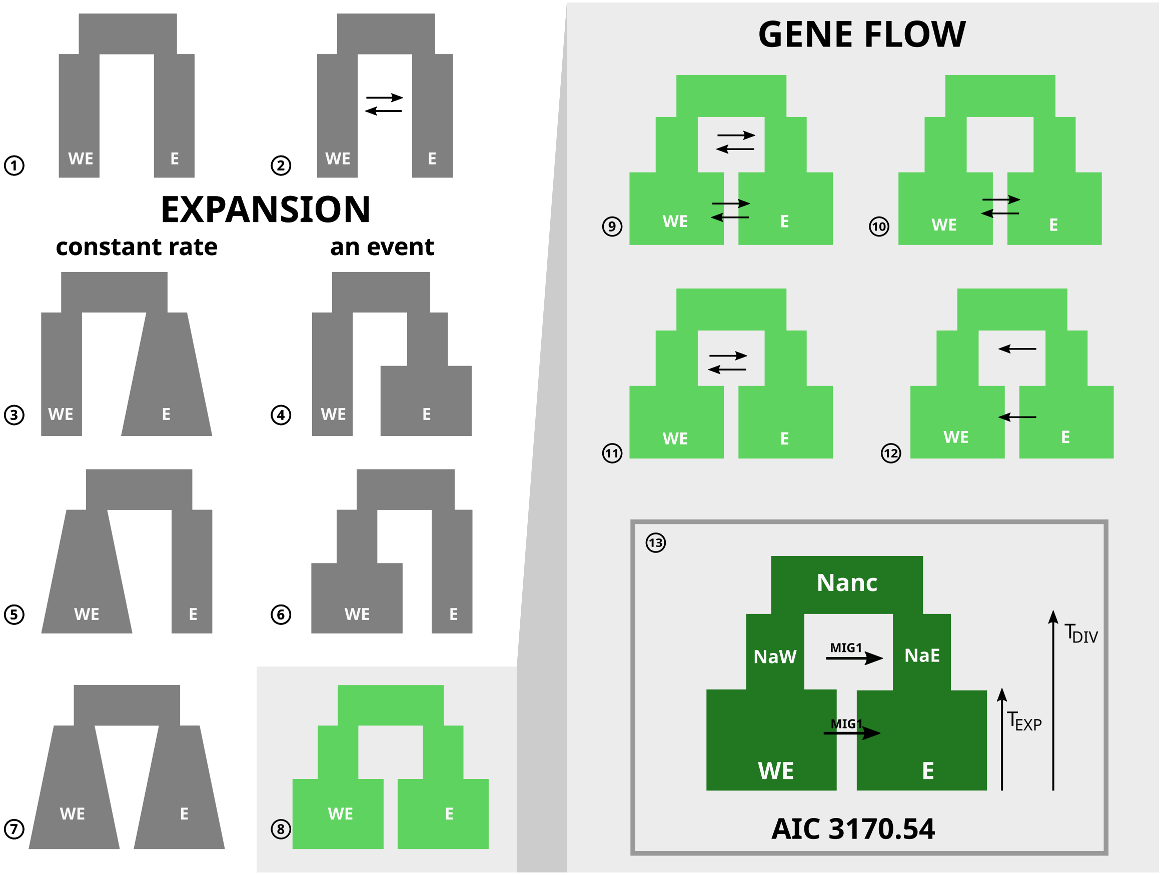

Supplementary Information 10: Figure S6. The demographic scenarios tested with FASTSIMCOAL. The scenarios are based on two main population groups, WE and E. The search parameters are in the Supplementary Information 11: Table S5. Nanc – ancestral effective population size; NaW and NaE ancestral effective population size for WE and E, respectively, before de expansion. WE and E currently effective population size. T_{EXP} – time of expansion in generations, and T_{DIV} – time of divergence in generations. MIG – unidirectional migration from WE to E.

Supplementary Information 11: Table S5. Search range for parameters used in fastSimcoal demographic scenarios.

Parameter	Distribution	min	max
E	uniform	100	100,000
WE	uniform	100	100,000
Nanc	uniform	10	10,000
NaW	uniform	10	10,000
NaE	uniform	10	10,000
TEXP	uniform	100	100,000
T_{DIV}	uniform	1000	100,000
MIG1	*Log*-uniform	0.000001	0.001
MIG2	*Log*-uniform	0.000001	0.001

The scenarios are based on the two main groups of populations, WE and E. Nanc – ancestral effective population size; NaW and NaE ancestral effective population size for WE and E, respectively, before de expansion. WE and E currently effective population size. T_{EXP} – time of expansion in generations, and T_{DIV} – time of divergence in generations. MIG1 – unidirectional migration from WE to E and MIG2 – unidirectional migration from E to WE.
